# Nutrients, Phytochemicals and In Vitro Disease Prevention of *Nephelium hypoleucum* Kurz Fruit

**DOI:** 10.3390/nu15040950

**Published:** 2023-02-14

**Authors:** Linh Khanh Luu, Sirinapa Thangsiri, Yuraporn Sahasakul, Amornrat Aursalung, Woorawee Inthachat, Piya Temviriyanukul, Nattira On-Nom, Chaowanee Chupeerach, Uthaiwan Suttisansanee

**Affiliations:** Food and Nutrition Academic and Research Cluster, Institute of Nutrition, Mahidol University, Salaya, Phuttamonthon, Nakhon Pathom 73170, Thailand

**Keywords:** antioxidant activities, aril, enzyme inhibition, non-communicable diseases, nutritional composition, organic acids, pericarp, phenolics, proximate analysis, seeds

## Abstract

*Nephelium hypoleucum* Kurz is an evergreen tree in the *Sapindaceae* family, mostly found in the forests of some Southeast Asia countries, especially Thailand. The lack of biological information regarding this tree has led to inappropriate agricultural management, conservation and utilization. Thus, this study aims to examine the nutritional composition, organic acid and phenolic profiles and in vitro health properties through several key enzyme inhibitions against some civilization diseases including Alzheimer’s disease (β-secretase (BACE-1), butyrylcholinesterase (BChE) and acetylcholinesterase (AChE)), obesity (lipase), hypertension (angiotensin-converting enzyme (ACE)) and diabetes (dipeptidyl peptidase-IV (DPP-IV), α-amylase and α-glucosidase) on the aril (flesh) part of *N. hypoleucum* Kurz fruit. The remaining fruit parts including the pericarp (peel) and seed were also assessed as sources of potential phenolics as well as key enzyme inhibitors. As results, carbohydrate (17.18 g) was found to be a major source of energy (74.80 kcal) in the aril (100 g fresh weight), with trace amounts of protein (0.78 g) and fat (0.32 g). The fruit aril also contained high insoluble dietary fiber (5.02 g) and vitamin C (11.56 mg), while potassium (215.82 mg) was detected as the major mineral. Organic acid profile indicated that the aril was rich in citric acid, while the phenolic profile suggested predominant quercetin and kaempferol. Interestingly, high gallic acid contents were detected in both pericarp and seed, with the latter 3.2-fold higher than the former. The seed also possessed the highest total phenolic content (TPC, 149.45 mg gallic acid equivalent/g dry weight), while total anthocyanin content (TAC, 0.21 mg cyanidin-3-*O*-glucoside equivalent/g dry weight) was only detected in pericarp. High TPC also led to high enzyme inhibitory activities in seed including BACE-1, AChE, BChE, ACE, DPP-IV and α-glucosidase. Interestingly, aril with the highest α-amylase inhibition suggested strong inhibitory distribution, predominantly from quercetin and kaempferol. Lipase inhibitory activities were only detected in the aril and pericarp, suggesting the biological function of these two phenolics and possibly anthocyanins.

## 1. Introduction

*Nephelium hypoleucum* Kurz, belonging to the *Sapindaceae* family, is a wild evergreen plant growing in the forests of Southeast Asia, including Thailand. This plant is in the same family as lychee (*Litchi chinensis* S.), rambutan (*Nephelium lappaceum* L.) and longan (*Dimocarpus longan* L.), and their fruits share similar physical characteristics. The fruit of *N. hypoleucum* Kurz, also known as wild lychee, is edible with a lychee-like odor but smaller in size (about 1–2 cm). The flesh is opaque white with a sweet and sour taste and often consumed fresh with a fermented fish dipping sauce, salted fish sauce or sweet fish sauce. The plant grows naturally in the forest and is not planted commercially for harvesting, with consumption limited to locals. As a result, this plant is in danger of extinction.

Being unpopular, limited information on nutritional and health properties of *N. hypoleucum* Kurz was available. Only two previous reports on *N. hypoleucum* Kurz were found [1,2]. The floral honey from *N. hypoleucum* Kurz exhibited high total phenolic contents and antioxidant capacity [1], while the whole fruit showed potential as a source of total phenolics and flavonoids [2]. Other indigenous plants in the *Sapindaceae* family have several health benefits and contain abundant nutrients and phytochemicals [3,4,5,6,7], with longan reported to have anti-inflammatory, antioxidant and anti-tyrosinase activities [3,4,5]. Despite limited information concerning health benefits, previous studies on related indigenous plants in the same family suggested that *N. hypoleucum* Kurz might also possess advantageous medicinal applications and contain distinct bioactive compounds with high nutritive values.

Apart from the aril (flesh), the remaining fruit parts from *N. hypoleucum* Kurz including pericarp (peel) and seed were investigated to determine whether they possessed any useful health-promoting properties or were sources of phytochemicals. *N. hypoleucum* Kurz grows in the forest and pesticide contamination was negligible. The fruit pericarp is reddish pink and may be a rich source of anthocyanins. The fruit is small and the aril is strongly attached to the seed, making separation difficult. The seed was also investigated for its health effects. The pericarp and seed from fruits in the same family were previously reported to possess bioactive compounds with potential health benefits. Pericarp extract of rambutan contained high contents of phenolics, demonstrating potential antioxidant properties [6], while lychee seed and fruit possessed many bioactivities such as hypoglycemic, anti-bacterial, anti-tyrosinase, anti-cancer, anti-hyperlipidemic, anti-platelet and anti-viral properties [7,8].

This study investigated the nutritional composition, bioactive compounds (phenolics and organic acids) and in vitro health properties against non-communicable diseases (NCDs) shown by key enzyme inhibitions in the aril part of *N. hypoleucum* Kurz fruit. Enzyme inhibition reduced the risk of NCD occurrence including Alzheimer’s disease (β-secretase (BACE-1), butyrylcholinesterase (BChE) and acetylcholinesterase (AChE)), obesity (lipase), hypertension (angiotensin-converting enzyme (ACE)) and diabetes mellitus (dipeptidyl peptidase-IV (DPP-IV), α-glucosidase and α-amylase). Other fruit parts including the pericarp and seed were also investigated for phytochemical contents and health-promoting activities. These findings present valuable knowledge for further functional food development and support the consumption of *N. hypoleucum* Kurz as a healthy fruit. This knowledge will also support cultural management, leading to future sustainable conservation and utilization of *N. hypoleucum* Kurz.

## 2. Materials and Methods

### 2.1. Sample Selection, Preparation and Extraction

**Figure 1:**
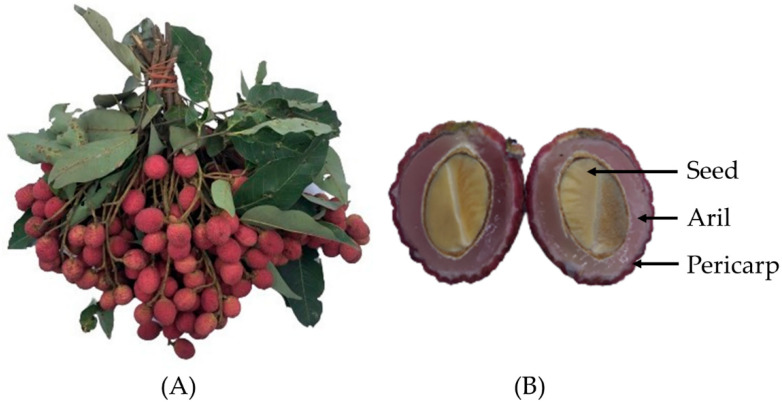
(**A**) A bunch of *Nephelium hypoleucum* Kurz fruit and (**B**) different parts of the fruit including aril (edible flesh), pericarp (peel) and seed.

The fruits of *N. hypoleucum* Kurz were collected from Ban Phra sub-district, Mueang district, Prachinburi province, Thailand (14°07′28.7″ N, 101°24′18.7″ E) in May, 2021 (120–140 days after flowering). A bunch contains about 10–15 fruits or more (Figure 1A). The shape of the fruit is round to elliptic with red color, and it possesses densely warty skin. The fruit size was measured using a digital vernier with a 0.01 mm/0.0005″ measuring range (Protronics Co., Ltd., Pathum Thani, Thailand) and it was found that the fruit exhibited 1.60 ± 0.09 cm in diameter and 2.08 ± 0.19 cm in length (*n* = 10). The samples were identified and authenticated by Dr. Sunisa Sangvirotjanapat (Mahidol University, Nakhon Pathom, Thailand) according to a reliable reference [9]. Dry samples were sent to Sireeruckhachati Nature Learning Park, Mahidol University (Nakhon Pathom, Thailand) for collection with assigned voucher specimen as PBM-005647.

All fresh samples were cleaned using deionized water before air-drying at room temperature for 2–3 h. The samples were then collected into three parts: aril (flesh), pericarp (peel) and seed (Figure 1B). Fresh aril was divided into two parts, one as fresh sample for nutritive values analysis and the other underwent freeze-drying utilizing a PL9000 freeze dryer (Heto Lab Equipment, Allerod, Denmark) for other experiments along with pericarp and seed. A Phillips 600 W series grinder (Phillips Electronics Co., Ltd., Jakarta, Indonesia) was used to grind the dry samples into powder. The powdery samples were then placed in vacuum aluminum foil bags and stored at −20 °C for further analysis.

Colors of fresh and dry fruits (aril, pericarp and seed) were examined utilizing a ColorFlex EZ spectrophotometer (Hunter Associates Laboratory, Reston, VA, USA) and presented as CIELAB units (L* for dark (−) to white (+), a * for green (−) to red (+) colors, and b * for blue (−) to yellow (+) colors), as shown in Supplementary Table S1. A Halogen HE53 moisture analyzer (Mettler-Toledo AG, Greifensee, Switzerland) was used for analyzing the moisture content of dry sample, as shown in Supplementary Table S2.

The extraction of the sample was carried out based on the previous study [2] with some modifications as follows. Briefly, the solvent containing 80% (*v/v*) ethanol (15 mL) was added to the powdery sample (3 g), and the reaction was shaken in a WNE45 temperature-controlled water bath shaker (Memmert GmBh, Eagle, WI, USA) at 30 °C for 4.5 h. The extractants were then collected by centrifugation at 3800× *g* for 10 min utilizing a Hettich^®^ ROTINA 38R refrigerated centrifuge (Andreas Hettich GmbH, Tuttlingen, Germany) before filtering through a 0.22 µm polyethersulfone (PES) syringe filter. The filtrates were kept in a freezer at −20 °C until analysis.

### 2.2. Determination of Nutrients

The standard protocols of Association of Official Analytical Chemists (AOAC) and American Association of Cereal Chemists (AACC) were employed for analysis of nutritional compositions including energy, moisture, protein, carbohydrate, fat, ash, sugar (glucose, fructose and sucrose), dietary fiber (soluble dietary fiber, insoluble dietary fiber and total dietary fiber), vitamins (B1, B2, B3, B5, B6, B7, B9 and B12) and minerals (Ca, Na, P, Mg, Fe and Zn) in the aril part of the fruit as previously described [10,11,12]. The experiments were performed at the Institute of Nutrition, Mahidol University under the Accredited Laboratory with the international standard for laboratory quality systems (ISO/IEC 17025: 2017).

Moisture content of fresh sample was examined utilizing a Memmenrt UNE 500 hot air oven (Memmert GmBh, Eagle, WI, USA) as a drying instrument. The temperature requirement was set at 95–105 °C with heat-dispersed equipment until the weight of sample was unchanged. The moisture content was calculated as the missing weight during the process (AOAC (2019) 925.45) using Equation (1).

(1) $$\%\;(w/w)\;\mathrm{Moisture}=\frac{W_{1}-W_{2}\;}{W1}\times 100$$

where W_1_ is a weight of a sample before evaporation (g) and W_2_ is a weight of a sample after evaporation (g).

Protein content was determined based on the Kjeldahl method by hydrolyzing protein using concentrated sulfuric acid (H_2_SO_4_) to generate ammonium sulfate ((NH_4_)_2_SO_4_). Then, strong alkaline was used to separate ammonia (NH_3_) from ammonium sulfate. Ammonia was then distilled and titrated with standard acid to collect total nitrogen. The protein content was calculated according to AOAC (2019) 991.20 by multiplying the amount of nitrogen (%) with a standard conversion factor of 6.25 using Equations (2) and (3).

(2) $$\%\;\mathrm{Total}\;\mathrm{nitrogen}=\frac{\left(V_{1}-V_{2}\right)\times \;M\;\times 14\;}{W\;\times 10}$$

% Crude Protein = % total nitrogen × 6.25,(3)

where V_1_ is a volume of an acid standard for titration of a sample (mL), V_2_ is a volume of an acid standard for titration of a reagent blank (mL), M is a molarity of a HCl standard and W is a weight of a sample portion or a standard (g).

Fat content was examined utilizing acid hydrolysis followed by petroleum ether extraction (AOAC (2019) 922.06) using a HT1043 Soxhlet system (Foss Tecator, Hoganas, Sweden) for 6 h. Crude fat was calculated using Equation (4).

(4) $$\%\;\mathrm{Crude}\;\mathrm{fat}=\frac{\left(W_{2}-W_{1}\right)\times 100\;}{S}$$

where W_1_ is a weight of empty flask (g), W_2_ is a weight of flask and extracted fat (g) and S is a weight of the sample (g).

Ash was determined through incineration in a CWF 1100 muffle furnace (Carbolite Gero Ltd., Hope Valley, UK) at 550 °C. The remaining residue was weighed and expressed as the ash content, following AOAC (2019) 930.30.

Total carbohydrate content was determined based on the contents of protein, fat, moisture and ash using Equation (5). Energy was calculated using Equation (6).

Total carbohydrate (g) = 100 − fat (g) − protein (g) − moisture content (g) − ash (g)(5)

Energy (kcal) = (total carbohydrate × 4) + (protein × 4) + (fat × 9)(6)

Sugars consisting of fructose, glucose and sucrose were examined using an ultra-fast liquid chromatography (UFLC) system (Shimadzu Corporation, Kyoto, Japan) equipped with an Alltech^®^ model 800 evaporative light scattering detector (ELSD) (BUCHI Corporation, New Castle, DE, USA) and a 5 μm Shodex Asahi Pak NH2P-50 4E column (250 × 4.6 mm, Shodex Group, Kanagawa, Japan). Sugars were separated utilizing 76% (*v/v*) acetonitrile and a flow rate of 1.0 mL/min as an isocratic solvent system (AOAC (2019) 980.13).

Total dietary fiber (TDF) is a sum of soluble dietary fiber (SDF) and insoluble dietary fiber (IDF), which were determined based on the enzymatic gravimetric method. The soluble and insoluble dietary fiber were examined according to AOAC (2019) 991.19 and 991.42, respectively. The sample was digested with heat-stable α-amylase, protease and amyloglucosidase. After the IDF was filtered, the residue was washed with warm deionized water. The washing water and filtrate were combined and precipitated with ethanol in order to determine the SDF content.

Vitamin B1 (thiamine) and B2 (riboflavin) extractions and determinations were carried out according to the protocol based on AOAC (2019) 942.23 and 970.65 [13], respectively. The HPLC system consisted of a Luna^®^ C18(2) 100 Å column (5 μm, 250 × 4.6 mm, Phenomenex, Torrance, CA, USA), a LC-20AT pump (Shimadzu Scientific Instrument, Columbia, MD, USA) and an FP-920 fluorescence detector (JASCO International Co., Ltd., Tokyo, Japan). An isocratic solvent system consisting of 50% (*v*/*v*) methanol and a 1.0 mL/min flow rate was set up for a separation of vitamin B1 and B2.

Vitamin B3 (niacin) extraction and determination were carried out following method based on AOAC (2019) 961.14. The HPLC system consisted of a Luna^®^ C8(2) 100 Å column (5 μm, 250 × 4.6 mm, Phenomenex, Torrance, CA, USA), a variable wavelength detector (VWD, 1100 series G1314B, Agilent Technologies, Santa Clara, CA, USA) and a pump (1200 series G1310A isocratic pump, Agilent Technologies, Santa Clara, CA, USA). An isocratic solvent system consisting of 15% (*v*/*v*) methanol and a 1.0 mL/min flow rate was set up for a separation of vitamin B3.

Microbiological methods were used to determine the contents of vitamins B5 (pantothenic acid), B6 (pyridoxine), B7 (biotin), B9 (folic acid) and B12 (cobalamin). The principle is based on the growth of microorganisms depending on the presence of vitamins in the culture media (using culture media with complete nutrition except for the vitamin to be determined). Samples preparation and extraction techniques using acid/alkaline hydrolysis with elevated temperature were according to the protocol of each vitamin. Culture media was added to the serial dilution of vitamin standard solutions or unknown samples, and a certain microorganism was inoculated and incubated. Microorganism growth was measured by turbidimetry using a Jenway™ Model 7315 UV/Visible Single Beam spectrophotometer (Bibby Scientific Ltd., Stone, UK.) at wavelength 620 nm, except for vitamin B9 at 630 nm. In-house methods based on AOAC (2019) were used unless otherwise stated. Vitamin B5 (AOAC 960.46 and 945.74 using *Lactobacillus plantarum*-ATCC No.8014), B6 (AOAC 961.15 using *Saccharomyces Carlsbergensis*), B7 (AOAC (1980) Ch 43.150–43.158 using *Lactobacillus plantarum* ATCC No.8014 [10]), B9 (960.46, 2004.05, and AACC method 86–47 [12] using *Lactobacillus case*i-ATCC No.7469), and B12 (960.46 and 952.20 using *Lactobacillus leichmannii*-ATCC No. 7830) were examined.

Vitamin C content was examined using HPLC analysis as indicated in a previous report [14]. The HPLC system consisted of a Zorbax ODS column (5 µm, 250 × 4.6 mm from Agilent Technologies, Santa Clara, CA, USA), a UV/Vis detector (UV-975, JASCO International Co., Ltd., Tokyo, Japan) and a Waters 515 pump (Waters Corporation, Milford, MA, USA). An isocratic solvent system consisting of 0.5% (*v/v*) KH_2_PO_4_ (adjusted pH to 2.5 with H_3_PO_4_) and a 0.8 mL/min flow rate was set up for a separation of vitamin C.

An S Series atomic absorption spectrometer (AAS) (Thermo Electron Corporation, Cambridge, UK) was employed to analyze the presence of calcium (Ca), sodium (Na) and potassium (K) (AOAC (2019) 985.35). Furthermore, an Optima 4200 DV inductively coupled plasma optical emission spectrophotometer (ICP-OES) (PerkinElmer^®^, Massachusetts, USA) was utilized to examine phosphorus (P), magnesium (Mg), zinc (Zn) and iron (Fe) (AOAC (2019) 984.27).

### 2.3. Determination of Organic Acids

Organic acids were analyzed utilizing reversed-phase high-performance liquid chromatography (RP-HPLC) using a well-established protocol as previously reported [15] with modifications as follows. The powdery sample (100 mg) was dissolved in 5 mL Milli-Q water (18.2 MΩ·cm resistivity at 25 °C) before incubating in a WNE45 series temperature-controlled water bath shaker (Memmert GmBh, Eagle, WI, USA) at 95 °C for 15 min. The mixture was then centrifuged at 3800× *g* for 5 min utilizing a Hettich^®^ ROTINA 38R refrigerated centrifuge (Andreas Hettich GmbH, Tuttlingen, Germany). The supernatants are then collected, while the remaining residue was re-extracted three times. The supernatants were pooled and filtered through 0.22 µm nylon syringe filter. The filtrate (10 µL) was then loaded into 250 × 4.6, 5 µm HyperSil Gold aQ columns (Thermo Fisher Scientific, Bremen, Germany) connected to a Waters Alliance HPLC-e2695 series and a diode array detector (DAD) (Waters Corporation, Milford, MA, USA). An isocratic solvent system consisting of 50 mM phosphate buffer (pH 2.8) and a 0.7 mL/min flow rate was set up for a separation of organic acids. A total run time was 20 min, while organic acids were visualized using a UV detection at 214 nm. The commercial standards including oxalic acid (≥99% RT), formic acid (98.0–100% assay), ascorbic acid (99% assay), acetic acid (≥99% assay), malic acid (≥99% assay), citric acid (≥99.5% assay), succinic acid (≥99% assay) and propionic acid (≥99.5% assay) were purchased from Sigma-Aldrich (St. Louis, MO, USA). The HPLC chromatograms of organic acid standards and samples were shown in Supplementary Figure S1.

### 2.4. Determination of Phenolic Profile

Phenolic profiles of aril, pericarp and seed of *N. hypoleucum* Kurz were performed on a liquid chromatography-electrospray ionization tandem mass spectrometry (LC-ESI-MS/MS) utilizing well-established protocols, parameters and validations according to the previous literature [16,17]. Briefly, the powdery samples were extracted with acidic methanol before loading onto an Accucore RP-MS column (a 2.1 mm × 100 mm, 2.6 μm column from Thermo Fisher Scientific, Bremen, Germany), which was connected to the LC-ESI-MS/MS system consisting of a Dionex Ultimate 3000 series ultrahigh-performance liquid chromatographer (UHPLC), a diode array detector, a TSQ Quantis Triple Quadrupole mass spectrometer (MS) and a Chromeleon 7 chromatography data system (Thermo Fisher Scientific, Bremen, Germany). Phenolics were separated using a gradient mobile phase consisting of acetonitrile (solvent A) and 0.1% (*v*/*v*) formic acid in Milli-Q water (18.2 MΩ·cm resistivity at 25 °C, solvent B) with 0.5 mL/min flow rate as follows: 10–80% solvent A and 90–20% solvent B at time 0.0–8.0 min; 80–10% solvent A and 20–90% solvent B at time 8.0–8.1 min; 10% solvent A and 90% solvent B at time 8.1–10.0 min.

The commercially available phenolic standards including caffeic acid (>98.0% HPLC, T), apigenin (>98.0% HPLC), 3,4-dihydroxybenzoic acid (≥97% T), genistein (>98.0% HPLC), hesperidin (>90.0% HPLC, T), (−)-epigallocatechin gallate (>98.0% HPLC), 4-hydroxybenzoic acid (>99.0% GC, T), kaempferol (>97.0% HPLC), *p*-coumaric acid (>98.0% GC, T), chlorogenic acid (>98.0% HPLC, T), naringenin (>93.0% HPLC, T), ferulic acid (>98.0% GC, T), cinnamic acid (>98.0% HPLC), syringic acid (>97.0% T), luteolin (>98.0% HPLC), sinapic acid (>99.0% GC, T), myricetin (>97.0% HPLC) and quercetin (>98.0% HPLC, E) were purchased from Tokyo Chemical Industry (Tokyo, Japan); rosmarinic acid (≥98% HPLC) and vanillic acid (≥97% HPLC) were received from Sigma-Aldrich (St. Louis, MO, USA); gallic acid (97.5–102.5% T), galangin (≥98.0% HPLC) and rutin (≥94% HPLC) were from Wuhan ChemFaces Biochemical Co., Ltd. (Hubei, China); isorhamnetin (≥99.0% HPLC) was from Extrasynthese (Genay, France). The LC-ESI-MS/MS chromatograms of phenolic standards and samples are shown in Supplementary Figure S2.

Total phenolic contents (TPCs) of the extract from Section 2.1 were determined utilizing Folin–Ciocalteu’s phenol reagent with a well-established protocol as reported in a previous study [18] without any modification. Gallic acid (0–200 µg/mL), a standard for TPC assay, was used to generate a calibration curve, Equation (7):

*y* = 0.0067*x* + 0.0405, (7)

where *y* is an absorbance and *x* is a gallic acid concentration. The results were present as milligrams of gallic acid equivalent (GAE)/g dry weight (DW) [19].

Total flavonoid contents (TFCs) of the extract from Section 2.1 were determined utilizing aluminum chloride colorimetric assay with a well-established protocol as reported in a previous study [20] without any modification. Quercetin (0–100 µg/mL), a standard for TFC assay, was used to generate a calibration curve Equation (8),

*y* = 0.0067*x* + 0.0405, (8)

where *y* is an absorbance and *x* is a quercetin concentration. The results were present as mg quercetin equivalent (QE)/g DW [21].

Total anthocyanin contents (TACs) were performed using pH differential method as previously reported [22] with some modifications as follows. The powdery sample (500 mg) was dissolved in 0.4 M sodium acetate (pH 4.5, 5 mL) and sonicated for 20 min using an ultrasonic cleansing bath (Branson Ultrasonics TM M series, Branson Ultrasonics Corp., Brookfield, CT, USA). The extract was then filtered through 0.45 µm polytetrafluoroethylene (PTFE) syringe filter (sample A, pH 4.5). To the sample A, 37% (*v/v*) HCl was added at a ratio of 4:1 (sample A:HCl) to prepare sample B (pH 1.0). Sample A was diluted by adding 0.4 M sodium acetate (pH 4.5) to be in the range of a linear standard curve (OD_520_ = 0.042 − 0.199). Sample B was diluted by adding 0.025 M KCl (pH 1.0) to be in the range of a linear standard curve (OD_520_ = 0.095 − 1.845). Both mixtures were detected at the wavelength of 520 and 700 nm by a Synergy^TM^ HT 96-well UV-visible microplate reader and a Gen 5 data analysis software (BioTek Instruments, Inc., Winooski, VT, USA). A standard for TAC assay was cyanidin-3-*O*-glucoside (2–60 µg/mL), and the results were presented as milligrams of cyanidin-3-*O*-glucoside equivalent (C3GE)/g DW, calculated using the Equation (9) as follows.

(9) $$\mathrm{TACs}=\frac{A\;\times \;\mathrm{MW}\;\times \;\mathrm{DF}\;\times 1000\;}{\varepsilon \;\times \;l\;\times \;C}$$

where A = (OD_520_ − OD_700_)_pH 1_._0_ − (OD_520_ − OD_700_)_pH 4.5_, DF = dilution factor, MW (molecular weight) = 449.2 g/mol for cyanidin-3-*O*-glucoside; 1000 = factor for conversion from g to mg, l = pathlength (cm), ε (extinction coefficient) = 26,900 L.mol^–1^. cm^–1^ for cyanidin-3-*O*-glucoside and C = concentration of the extract (mg/mL).

### 2.5. Determination of Antioxidant Activities

Three antioxidant assays including ferric ion reducing antioxidant power (FRAP), 2,2-diphenyl-1-picrylhydrazyl (DPPH) radical scavenging and oxygen radical absorbance capacity (ORAC) assays were performed as previously described [18] using the extract from Section 2.1. Briefly, FRAP assay consisted of FRAP reagent containing 2,4,6-tri(2-pyridyl)-*S*-triazine, acetate buffer and FeCl_3_·6H_2_O solution and an end-point detection at 600 nm, while the DPPH radical scavenging assay employed DPPH radical solution as reagent and an end-point detection at 520 nm. On the other hand, the ORAC assay used 2,2′-azobis(2-amidinopropane) dihydrochloride and sodium fluorescein as the main reagents with kinetical detection at 485 nm excitation wavelength and 528 nm emission wavelength. All reactions were visualized using a Synergy^TM^ HT 96-well UV-visible microplate reader and a Gen 5 data analysis software (BioTek Instruments, Inc., Winooski, VT, USA). Trolox was used as a standard, and the extract concentrations in all antioxidant assays were in the range of the Trolox standard curve. The results were presented as micromoles of Trolox equivalent (TE)/g DW.

### 2.6. Determination of Enzyme Inhibitory Activities

Enzyme inhibitory activities were performed using the extract from Section 2.1. Inhibitions of the key enzymes including BACE-1, AChE, BChE, lipase, ACE, DPP-IV, α-glucosidase and α-amylase were assessed using previously reported well-established protocols [20,23,24,25], as indicated in Table 1. The ACE inhibitory assay was adapted from a previous report [26] with some modifications. Briefly, a mixture of the substrate (3 mM *N*-hippuryl-His-Leu tetrahydrate, 90 µL), the enzyme (0.5 U/mL ACE from rabbit lung (≥2 units/mg protein), 9 µL) and the extract (150 µL) was incubated at 37 °C for 30 min. The reaction was stopped by adding 0.28 M NaOH (531 µL). Then, 20 mg/mL *O*-phthaldialdehyde (45 µL) as an indicator was added to the reaction and mixed well. The pH was adjusted to neutral by adding 3 M HCl (75 µL). The reaction was visualized using an excitation wavelength of 360 nm and emission wavelength of 485 nm. Kinetic inhibitory assays including lipase, α-glucosidase, α-amylase, DPP-IV, AChE and BChE, and end-point inhibitory assays including BACE-1 and ACE were visualized using a Synergy^TM^ HT UV–visible microplate reader and Gen 5 data analysis software (BioTek Instruments, Inc., Winooski, VT, USA). All chemicals and reagents used in Table 1 were purchased from Sigma-Aldrich (St. Louis, MO, USA).

The percentage of inhibition was calculated using Equation (10) as follows.

(10) $$\%\;\mathrm{inhibition}=(1-\;\frac{B-b}{A-a})\times 100$$

where *A* is an initial velocity (*V*_0_) of a reaction with an enzyme but without a fruit extract (control), *a* is a *V*_0_ of a reaction without an enzyme and a fruit extract (control blank), *B* is a *V*_0_ of a reaction with an enzyme and a fruit extract (sample), and *b* is a *V*_0_ of a reaction with a fruit extract but without an enzyme (sample blank).

### 2.7. Statistical Analysis

All experiments were analyzed in triplicate of three independent set of samples (*n* = 3). The results were shown as mean ± standard deviation (SD). Statistical analysis with significant differences at *p* < 0.05 was determined utilizing one-way analysis of variance (ANOVA) with Duncan’s multiple comparison. Principal component analysis and hierarchical cluster analysis of TPCs, TFCs, TACs, antioxidant potentials and enzyme inhibitory activities were determined using XLSTAT^®^ (Addinsoft Inc., New York, NY, USA).

## 3. Results

### 3.1. Nutrional Compositions

Nutritive values including energy, fat, protein, carbohydrate, TDF, IDF, SDF, total sugar (glucose, fructose and sucrose), ash, vitamins (B1, B2, B3, B5, B6, B7, B9, B12 and C) and mineral contents (Na, K, Ca, P, Mg, Fe and Zn) of the aril of *N. hypoleucum* Kurz fruit were analyzed utilizing AOAC and AACC standard protocols. Results are presented in Table 2 as per 100 g fresh weight (FW) and calculated as per 100 g dry weight (DW) using moisture content in an attempt to compare with those in the same family (lychees, rambutans and longans) that were previously reported.

The aril (per 100 g FW) contained total energy of 74.80 kcal, which came from protein of 0.78 g, fat of 0.32 g and carbohydrate of 17.18 g. Therefore, the energy mainly came from carbohydrate. The IDF content was higher than SDF by 8.8-fold and considered as the major component in TDF. Monosaccharides including glucose and fructose were predominant, with disaccharide content (sucrose) 2.1 to 2.5-fold lower. Ash, as the powdery residue after burning, was detected in extremely small amounts (0.56 g) and used to calculate mineral quantities. Vitamins B3 (0.28 mg) and B12 (0.37 mg) were detected in higher amounts than B1, B2, B5 and B6 (0.02–0.03 mg), while only 2 µg and 7.88 µg dietary folate equivalents (DFE) were detected in vitamins B7 and B9, respectively. Vitamin C was the highest (11.56 mg) among all examined vitamins. The main minerals detected were K (215.82 mg), Na (28.83 mg), Ca (18.88 mg), P (9.81 mg) and Mg (5.25 mg). Microminerals including Fe and Zn were detected at lower amounts ranging from 0.20 to 0.24 mg.

### 3.2. Organic Acid and Phenolic Profiles

The organic acid profile of the aril of *N. hypoleucum* Kurz fruit was analyzed by HPLC using eight organic acid standards including oxalic acid, formic acid, acetic acid, ascorbic acid, citric acid, succinic acid, malic acid and propionic acid (Table 3, Supplementary Figure S1). Only the aril, not the pericarp and seed, was examined in this experiment. This edible part of the fruit has a sour taste, and the organic profile offered useful information for further investigations on food development. Results indicated that the aril contained six organic acids including oxalic acid, formic acid, ascorbic acid, acetic acid, malic acid and citric acid, with the last predominant at up to 3413-fold higher than the others. Succinic acid and propionic acid were not detected.

The phenolic profiles of the aril, pericarp and seed of *N. hypoleucum* Kurz fruit were analyzed by LC-ESI-MS/MS using twenty-four phenolic standards including chlorogenic acid, caffeic acid, cinnamic acid, apigenin, *p*-coumaric acid, 3,4-dihydroxybenzoic acid, (−)-epigallocatechin gallate, ferulic acid, galangin, gallic acid, genistein, hesperidin, 4-hydroxybenzoic acid, kaempferol, isorhamnetin, luteolin, myricetin, naringenin, quercetin, rosmarinic acid, sinapic acid, rutin, syringic acid and vanillic acid (Table 4). Results indicated that the aril contained seven phenolics, predominantly quercetin, followed by kaempferol, gallic acid, luteolin, isorhamnetin, rutin and naringenin, respectively. Quercetin content in the aril was 1.5 to 3.9-fold higher than that found in the pericarp and seed, while kaempferol at 2.2-fold lower than quercetin was higher in the aril than in other fruit parts. The same seven phenolics were also detected in the pericarp, with gallic acid the main constituent. Luteolin and rutin at 9.3- and 163.7-fold lower than gallic acid, respectively, were the only phenolics detected in the pericarp and were higher than in other fruit parts. Only four phenolics including gallic acid, rutin, quercetin and naringenin were detected in the seed. Similar to the pericarp, gallic acid was the main phenolic detected in the seed at 3.2-fold higher than in pericarp and 24.1-fold higher than in aril. Naringenin in seed was found in lower amounts than gallic acid (106.6-fold lower) but higher than in pericarp (4.1-fold higher) and aril (30.6-fold higher).

With high gallic acid content, seed exhibited the highest TPC among the different fruit parts (8.1- and 17.3-fold higher than in pericarp and aril, respectively) (Table 4). The seed also possessed the highest TFC even though the phenolic profile suggested higher flavonoid contents in the aril. Thus, seed might contain particular flavonoids that were not included as phenolic standards in the LC-ESI-MS/MS analysis. As expected, only the pericarp contained anthocyanins since this fruit part is red.

### 3.3. Antioxidant Potentials

Antioxidant potentials of the aqueous ethanolic extract of the aril, pericarp and seed of *N. hypoleucum* Kurz fruit were analyzed utilizing DPPH radical scavenging, FRAP and ORAC assays (Table 5). The seed exhibited the highest antioxidant activities determined by all three assays followed by pericarp (9.9-, 6.7- and 1.3-fold lower in DPPH radical scavenging, FRAP and ORAC activities, respectively) and aril (21.8-, 17.2- and 5.7-fold lower in DPPH radical scavenging, FRAP and ORAC activities, respectively).

### 3.4. Enzyme Inhibitory Properties

The in vitro enzymatic inhibitory activities of the aqueous ethanolic extract of the aril, pericarp and seed of *N. hypoleucum* Kurz fruit were analyzed spectrophotometrically (Table 6). Control of key enzymes can ameliorate the occurrence of some NCDs, and results suggested that these fruit extracts could reduce the risk of NCDs.

Lipase is a lipid-degrading enzyme and inhibition of lipase slows down the rate of fatty acid absorption into the body as one of the medicinal treatments for obesity [28]. At an extract concentration of 8 mg/mL, only the aril and pericarp inhibited lipase at 51.59 and 59.48%, respectively, while no lipase inhibition was observed in seed using the same extract concentration.

Diabetes mellitus (DM) occurrence is related to increase in blood glucose. The key enzymes involved in DM including α-amylase, α-glucosidase and DPP-IV were studied in this research. Inhibition of the carbohydrate hydrolyzing enzymes, α-amylase and α-glucosidase, can delay carbohydrate degradation and slow down the rate of glucose absorption, leading to control of DM [29,30]. Inhibition of DPP-IV regulates insulin secretion, resulting in reduced postprandial and fasting hyperglycemia [31]. Results indicated that all fruit extracts inhibited α-amylase ranging from 35.05 to 68.57% at an extract concentration of 8 mg/mL. The aril exhibited 1.8- and 2.4-fold higher α-amylase inhibitory activities than seed and pericarp, respectively. Stronger inhibition was observed in the α-glucosidase assay (ranging 81.19–93.20%) even at lower extract concentration of 1 mg/mL. The seed exhibited the highest α-glucosidase inhibitory activity at 1.1-fold higher than in the aril and pericarp. An extract concentration of 1 mg/mL seed exhibited 2.4-fold higher DPP-IV inhibitory activity than the aril, while no inhibition was observed in the pericarp.

Cholinesterase enzymes (ChEs) including AChE and BChE are neurotransmitter (acetylcholine) degrading enzymes that lead to decline in brain cognitive function that eventually develops into AD. Formation of β-amyloid peptides by BACE-1 also causes deposition of amyloid plaque in the brain as one of the AD developments. Thus, inhibition of these enzymes can reduce the risk of AD occurrence [32,33]. Using an extract concentration of 8 mg/mL, all fruit extracts inhibited both AChE (51.33–84.56% inhibition) and BChE (32.57–90.87% inhibition). In both assays, seed exhibited higher AChE and BChE inhibitory activities than the aril and pericarp (1.4 to 1.6-fold higher in the AChE reaction and 1.8 to 2.8-fold higher in the BChE reaction). Using the same extract concentration of 8 mg/mL, only seed exhibited BACE-1 inhibitory activity at 66.33% inhibition, while no inhibition was detected in the aril and pericarp.

Angiotensin-converting enzyme (ACE) is the key enzyme related to hypertension. This enzyme degrades angiotensin I to angiotensin II, an octapeptide that causes the muscular walls of small arteries to collapse, thereby increasing blood pressure [34]. Hence, inhibiting angiotensin II formation through ACE inhibition can control blood pressure. Results showed that all fruit extracts inhibited ACE activities at 65.79–85.18% using extract concentration of 0.8 mg/mL, with seed giving 1.2 to 1.3-fold higher inhibitory activities than aril and pericarp.

### 3.5. Principal Component Analysis (PCA) and Hierarchical Cluster Analysis (HCA)

**Figure 2:**
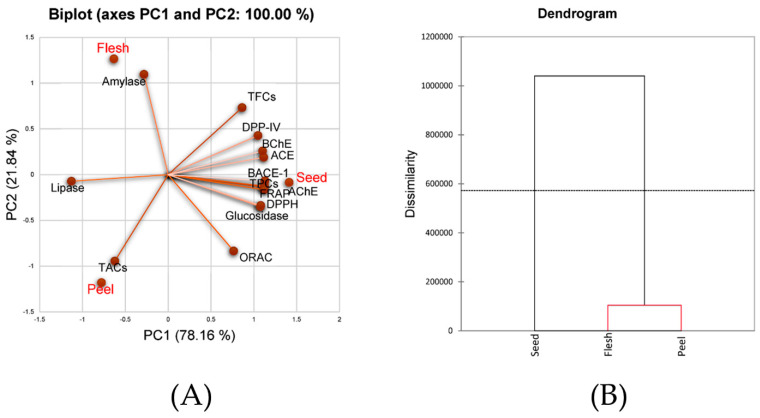
(**A**) Biplot of principal component analysis (PCA) generated from the observations including aril (flesh), pericarp (peel) and seed of *Nephelium hypoleucum* Kurz and variables including total phenolic contents (TPCs), total anthocyanin contents (TACs), total flavonoid contents (TFCs), antioxidant activities (DPPH radical scavenging, FRAP and ORAC activities) and enzyme inhibitory activities against lipase, dipeptidyl peptidase-IV (DPP-IV), α-glucosidase, α-amylase, angiotensin-converting enzyme (ACE), β-secretase (BACE-1), butyrylcholinesterase (BChE) and acetylcholinesterase (AChE); (**B**) dendrogram derived from the hierarchical cluster analysis (HCA) from the same data as PCA.

Data were subjected to principal component analysis (PCA), which can accurately convert large-scale data into interpretable figures. Mean phytochemical contents (TPCs, TFCs and TACs), antioxidant activities (through DPPH radical scavenging, ORAC and FRAP assays) and enzyme inhibitory activities (lipase, α-glucosidase, α-amylase, DPP-IV, ACE, AChE, BChE and BACE-1) were analyzed as variables, while aril, pericarp and seed were observations (Figure 2). 

The obtained biplots showed two axes including PC1 and PC2 (Figure 2A). Data representation as 78.16% on PC1 and 21.84% on PC2 resulted in 100%, indicating that the biplot interpreted the data with high accuracy. The seed was positioned together with almost all the tested variables including phytochemical contents (TPCs and TFCs), antioxidant activities (through DPPH radical scavenging, ORAC and FRAP assays) and enzyme inhibitory activities (α-glucosidase, DPP-IV, ACE, AChE, BChE and BACE-1). Results implied that seed had high contents of these tested variables. TPCs and TFCs but not TACs were located in close proximity to seed, suggesting that high enzyme inhibitory activities against α-glucosidase, DPP-IV, ACE, AChE, BChE and BACE-1 may result from phenolic distribution of flavonoids but not anthocyanins. By contrast, seed was located opposite to lipase, suggesting no relationship between seed and lipase inhibitory activities. Both aril and pericarp were positioned far away from most variables compared to seed, thereby indicating the uniqueness of seed as the source of bioactive agents of *N. hypoleucum* Kurz.

The hypothesis that the seed was unique compared to aril and pericarp was confirmed by hierarchical cluster analysis (HCA), which is typically used to determine the clustering of objects. Mean data of all variables were used in the analysis. The HCA data illustrated two clusters containing aril and pericarp as cluster 1 and seed as cluster 2 (Figure 2B), thereby confirming the hypothesis suggested by PCA.

## 4. Discussion

*N. hypoleucum* Kurz is a wild plant with sour fruits but limited information exists on its nutritional composition, phytochemicals and health properties. Historically, this plant was used as folk medicine by locals as a laxative and blood-dispersing agent. The plant grows in forested areas, and the fruits are only consumed by local people. To prevent extinction and promote fruit consumption and agricultural management, the nutritive values, phenolic compositions and in vitro health properties through inhibition of key enzymes relevant to the control of some NCDs were examined for the aril part of the fruit. The pericarp and seed were also examined for their phenolic and health-promoting properties as alternative potential future food sources, following the concept of zero food waste. Results indicated that (i) the aril was mainly composed of carbohydrates with high IDF, vitamin C and K, (ii) other than vitamin C, the sour taste of the aril might result from high citric acid content, (iii) among the three fruit parts, seed contained the highest TPC with gallic acid a predominant phenolic, while anthocyanins were only detected in the pericarp, (iv) high TPC in seed led to high antioxidant activities and most enzyme inhibitions and (v) the aril and pericarp contained lower TPCs with lipase inhibition >50%, while lipase inhibitory activity was not detected in seed.

Compared to other fruits in the same family (per 100 g DW, Table 2), the aril provided similar energy as rambutan but higher than lychee and longan [27]. The fruits in this family contain carbohydrates as a major nutritional component (89–92% DW), followed by protein (4–6% DW) and fat (0.5–1.7% DW). Based on the available data, *N. hypoleucum* Kurz exhibited 4.2 to 6.1-fold higher dietary fiber content than lychee and longan but 2-fold lower sugar content than lychee. *N. hypoleucum* Kurz also exhibited lower vitamin contents than other fruits in the same family, with vitamin C 2.9 to 5.4-fold lower than the other fruits. According to the Thai Recommended Daily Intakes (Thai RDIs) [35], 100 g of fresh *N. hypoleucum Kurz*. provides up to 22 and 19% of daily intakes for dietary fiber and vitamin C, respectively. Interestingly, *N. hypoleucum* Kurz contains 2.3 to 27.5-fold higher Na and 1.3 to 2.9-fold higher Ca than other fruits in the same family. However, 100 g of fresh fruit provided only 1.5 and 2.4% of Thai RDIs for Na and Ca, respectively, while K and Zn contents *in N. hypoleucum* Kurz were similar to other fruits. *N. hypoleucum Kurz* had lower P, Mg and Fe contents than other fruits in the same family (1.7 to 3.5, 2.5 and 1.4 to 2.9-fold lower, respectively).

Organic acids play an important economical role with several applications in food, pharmaceuticals and chemical processes. They are an authentic indicator because of their lower susceptibility during food processing and storage. The combination of organic acids and sugars contributes to the sensory quality of raw and processed fruits. Thus, quantitative analysis of the main organic acids is essential in the food and beverage industry to evaluate product quality. *N. hypoleucum* Kurz aril was high in citric acid, while smaller amounts of other organic acids were also detected. Citric acid was also predominantly found in various cultivars of rambutan [36], while tartaric acid and malic acid were mainly found in different cultivars of lychee [37]. Similarly, malic acid was also predominantly detected in longan [38]. Compared to other fruits in the same family, *N. hypoleucum* Kurz expressed higher amounts of citric acid [36,37,38], causing a sour taste.

When comparing the aril parts of fruits in the same family, TPC of *N. hypoleucum* Kurz aril (163.09 mg GAE/100 g FW) was compatible with 80% aqueous acetone extracts of 13 lychee cultivars harvested from Southern China (101.51–259.18 mg GAE/g FW) [39] but higher than 80% aqueous acetone extracts of 24 longan cultivars (22.09–132.47 mg GAE/100 g FW) [40]. Rambutan extracted with different solvents (water, 70% aqueous ethanol and 70% aqueous methanol) exhibited TPCs ranging from 1.45 to 3.10 mg chlorogenic acid equivalent/100 g DW [41]. *N. hypoleucum* Kurz pericarp exhibited lower TPC (18.53 mg GAE/g DW) than a 70% aqueous methanolic extract of lychee pericarp (24.6 mg GAE/g DW) [42], 95% (*v/v*) ethanolic extracted rambutan pericarp (42.3 mg GAE/g DW) [2] and 80% acidic aqueous ethanolic extracted longan pericarp (53.5 mg GAE/g DW) [43]. Interestingly, only *N. hypoleucum* Kurz pericarp contained anthocyanin (0.21 mg C3GE/g DW or 3.95 mg C3GE/100 g FW). This result corresponded to fruits in the same family with pink-reddish pericarp that were also reported to contain anthocyanins. Rambutan pericarp extracted with acidic aqueous ethanol (80% *v/v*) exhibited TAC of 181 mg C3GE/100 g FW [44], while acidic extracted lychee pericarp exhibited TAC of 75 mg C3GE/100 g FW [45]. However, lower TPCs in the aril and pericarp (8.66 and 18.53 mg GAE/g DW, respectively) and higher TPCs in the seed (149.45 mg GAE/g DW) of *N. hypoleucum* Kurz were recorded than for 95% aqueous ethanolic extract of its whole fruit (89.6 mg GAE/g DW), suggesting that most phenolics extracted from the whole fruit came from its seed [2]. *N. hypoleucum* Kurz seed exhibited higher TPC than an ultrasonic-assisted extraction of longan seed, with TPC of 48.92 mg GAE/g DW [46], 95% aqueous ethanolic extract of rambutan seed (43.5 mg GAE/g DW) [2] and 70% aqueous methanolic extract of lychee seed (17.9 mg GAE/g DW) [42]. These data suggested that *N. hypoleucum* Kurz seed was a significant source of phenolics.

High TPC in the seed of *N. hypoleucum* Kurz led to high antioxidant activities, with a strong correlation between phenolic content and antioxidant activities, concurring with previous reports [16,47]. Gallic acid, a predominant phenolic detected in seed, is a strong antioxidant with a half-maximal scavenging concentration (EC_50_) of 140 µmol/mL determined by DPPH radical scavenging assay [48]. Quantities of 320 µmol TE/mole antioxidant and 1.1 mol TE/mole antioxidant were detected in FRAP and ORAC assays, respectively [48]. This information suggested that gallic acid is the main phenolic causing high antioxidant activity in the seed of *N. hypoleucum* Kurz.

High TPC in seed also led to high inhibitory activities against α-glucosidase, DPP-IV, AChE, BChE, BACE-1 and ACE. Gallic acid was the predominant phenolic detected in seed and possibly responsible for these enzyme inhibitory activities. Gallic acid acts as a competitive α-glucosidase inhibitor, with half maximal inhibitory concentration (IC_50_) of 1.29 mM [49]. Its inhibitory strength was compatible to that of acarbose, a commercially available anti-diabetic drug that acts as a competitive α-glucosidase inhibitor with IC_50_ of 1.28 mM [49]. Gallic acid also inhibited DPP-IV with IC_50_ value of 4.65 µM, similar to diprotin A, a DPP-IV commercially available inhibitor (IC_50_ 4.21 µM) [50]. Gallic acid exhibited IC_50_ value of 3 mM against AChE [51], while its BChE inhibitory activity was 3-fold higher [52]. However, its inhibitory strength was less effective than galanthamine, a commercially available cholinesterase inhibitor, with IC_50_ values of 1.6 and 10.9 µM against AChE and BChE, respectively. The large amounts of gallic acid detected in seed possibly contributed to high AChE and BChE inhibitory activities. Gallic acid inhibited BACE-1 at 35% at a concentration of 100 µM [53] and disrupted Aβ_1-42_ accumulation [54]. Gallic acid was also reported to be a potential ACE inhibitor with IC_50_ value of 37.38 μg/mL and it clearly decreased the blood pressure of spontaneously hypertensive rats (SHR) [55]. Hypertension is associated with sodium levels in the body. Therefore, consuming *N. hypoleucum* Kurz as a low-sodium fruit can be advantageous, with fewer side effects than using anti-hypertensive drugs.

Despite exhibiting lower TPCs than seed, aril and pericarp inhibited lipase by >50% at 8 mg/mL, while none was detected in seed. Since gallic acid was a predominant phenolic in pericarp and seed, other phenolics possibly contributed to this inhibitory activity. Other than gallic acid, the main phenolics detected in aril and pericarp were flavonoids including quercetin, kaempferol and luteolin. Quercetin inhibited pancreatic lipase in a mixed-type non-competitive manner [56] with IC_50_ value of 6.1 μM [57]. Quercetin is considered a weaker inhibitor compared to orlistat, a competitive lipase inhibitor and an anti-obesity drug approved by the Food and Drug Administration (FDA), with IC_50_ value of 4.0 μM [57]. However, an in vivo study indicated that fat absorption was significantly reduced by pre-administration of 5 and 10 mg quercetin/kg body weight of rat [56]. Kaempferol was also previously reported to be a competitive inhibitor with IC_50_ value of 229.20 μM, which was 3.8-fold higher than orlistat (performed under the same inhibitory assay), indicating its weaker strength compared to inhibit lipase [58]. Scant information is available on lipase inhibitory activity of luteolin but reports suggested that luteolin inhibited lipase at 1.4-fold higher IC_50_ value than quercetin [59], suggesting weaker strength. These flavonoids exhibited lower lipase inhibitory strength than orlistat but high concentrations present in the aril and pericarp of *N. hypoleucum* Kurz were distributed to give high lipase inhibitory activities.

## 5. Conclusions

This is the first report on the nutritional compositions, bioactive compounds and in vitro health properties of *N. hypoleucum* Kurz fruit. The aril was found to be nutritious, with higher fiber content than other fruits in the same family. The aril also contained various types of vitamins and minerals as well as high amounts of citric acid, showing promise for future promotion of consumption and product development. Other than the edible part, pericarp and seed of *N. hypoleucum* Kurz were potential sources of phenolics with high antioxidant activities and health-related properties. The seed exhibited the highest antioxidant and most enzyme inhibitory activities, with high phenolic contents as predominantly gallic acid. However, further detailed investigations into the toxicity of these inedible plant parts are required to ensure their safety before development into other applications. Other than phenolics, it is possible that metal elements might be able to interfere with enzyme inhibitions and antioxidant tests. However, these experiments employed commercially available holoenzyme and FRAP reagent (Fe^3+^-TPTZ); this concern is void. Furthermore, more experiments on enzyme inhibitions should be performed and reported as IC_50_ values in order to compare the effectiveness with other previously reported extracts. This information would also be useful for further cell culture and in vivo studies.

## Figures and Tables

**Table 1:** The assay components including an enzyme, a substrate, an indicator, a fruit extract and a detection wavelength for enzyme inhibitory assays.

Assay	Assay Components				
	Enzyme	Substrate	Indicator	Extract	Detection Wavelength
Lipase	100 µL of 20 µg/mL lipase ^1^	50 μL of 0.2 mM DMPTB	10 µL of 16 mM DTNB	40 µL	412 nm
BChE	100 μL of 1.5 µg/mL BChE ^2^	50 μL of 0.4 mM BCh			
AChE	100 μL of 0.25 µg/mL AChE ^3^	50 μL of 0.32 mM ACh			
DPP-IV	50 µL of 0.02 U/mL DPP-IV ^4^	25 µL of 12 mM Gly-Pro-pNA + 100 µL Tris-HCl (pH 8)		25 µL	405 nm
α-Glucosidase	10 µL of 0.2 U/mL α-glucosidase ^5^	25 µL of 10 mM pNPG + 160 µL KPB (pH 7)		5 µL	
α-Amylase	100 µL of 50 mg/mL α-amylase ^6^	50 µL of 30 mM pNPM		50 µL	
BACE-1	BACE-1 FRET assay kit (Sigma-Aldrich, St. Louis, MO, USA) following manufacturer’s recommendations				λ_ex_ = 320 nm λ_em_ = 405 nm

^1^*Candida rugosa* lipase (type VII, ≥700 unit/mg); ^2^ equine serum BChE (≥10 units/mg); ^3^ *Electrophorus electricus* AChE (1000 units/mg); ^4^ recombinant human dipeptidyl peptidase-IV (≥10 units/mg); ^5^ *Saccharomyces cerevisiae* α-glucosidase (type I, ≥10 U/mg protein); ^6^ porcine pancreatic α-amylase (type VII, ≥10 unit/mg). DMPTB: 2,3-Dimercapto-1-propanol tributyrate; DTNB: 5,5′-dithiobis(2-nitrobenzoic acid); AChE: acetycholinesterase; ACh: acetylthiocholine; BChE: butyrylcholinesterase; BCh: butyrylthiocholine; DPP-IV: dipeptidyl peptidase-IV; Gly-Pro-pNA: Gly-Pro-*p*-nitroanilide hydrochloride; pNPG: *p*-nitrophenyl-α-D-glucopyranoside; KPB: potassium phosphate buffer; pNPM: *p*-nitrophenyl-α-D-maltohexaoside; BACE-1: β-secretase; FRET: fluorescence resonance energy transfer.

**Table 2:** Nutritional compositions of the aril part of *Nephelium hypoleucum* Kurz. compared to the reported literature on lychees (*Litchi chinensis* S.), rambutans (*Nephelium lappaceum* L.) and longans (*Dimocarpus longan* L.).

Nutrients	*Nephelium hypoleucum* Kurz		Lychees		Rambutans		Longans	
	per 100 g FW	per 100 g DW	per 100 g FW ^a^	per 100 g DW ^b^	per 100 g FW ^a^	per 100 g DW ^b^	per 100 g FW ^a^	per 100 g DW ^b^
**Energy (kcal)**	74.70 ± 0.68	396.61 ± 0.71	65.68 ^c^	388.64 ^c^	76.82	393.95	79.07 ^c^	387.60 ^c^
**Moisture (g)**	81.17 ± 0.21	0.00	83.1	0.00	80.5	0.00	79.6	0.00
**Protein (g)**	0.78 ± 0.01	4.14 ± 0.01	0.96	5.68	0.97	4.97	1.17	5.74
**Fat (g)**	0.32 ± 0.02	1.70 ± 0.12	0.16	0.95	0.14	0.72	0.11	0.54
**Carbohydrate (g)**	17.18 ± 0.21	91.19 ± 0.10	15.1 ^d^	89.35 ^d^	17.92	91.90	18.35 ^d^	89.95 ^d^
TDF (g)	5.59 ± 0.04	29.66 ± 0.56	1.20	7.10	NA	NA	1.00	4.90
SDF (g)	0.57 ± 0.03	3.00 ± 0.17	NA	NA	NA	NA	NA	NA
IDF (g)	5.02 ± 0.02	26.66 ± 0.40	NA	NA	NA	NA	NA	NA
**Total Sugar (g)**	10.22 ± 0.37	54.25 ± 2.58	17.95	106.21	NA	NA	NA	NA
Glucose (g)	3.76 ± 0.16	19.97 ± 1.07	NA	NA	NA	NA	NA	NA
Fructose (g)	4.59 ± 0.15	24.38 ± 1.06	NA	NA	NA	NA	NA	NA
Sucrose (g)	1.87 ± 0.06	9.91 ± 0.45	NA	NA	NA	NA	NA	NA
**Ash (g)**	0.56 ± 0.01	2.97 ± 0.02	0.65	3.85	0.46	2.36	0.73	3.58
**Vitamins**								
B1 (mg)	0.02 ± 0.00	0.11 ± 0.00	0.02	0.12	0.01	0.05	0.01	0.05
B2 (mg)	0.02 ± 0.00	0.11 ± 0.00	0.10	0.59	0.08	0.41	0.06	0.29
B3 (mg)	0.28 ± 0.03	1.46 ± 0.15	1.02	6.04	0.78	4.00	0.83	4.07
B5 (mg)	0.03 ± 0.01	0.13 ± 0.03	NA	NA	NA	NA	NA	NA
B6 (mg)	0.03 ± 0.00	0.16 ± 0.00	NA	NA	NA	NA	NA	NA
B7 (µg)	2.00 ± 0.00	10.62 ± 0.12	NA	NA	NA	NA	NA	NA
B9 (µg DFE)	7.88 ± 0.63	41.79 ± 2.86	NA	NA	NA	NA	NA	NA
B12 (mg)	0.37 ± 0.02	1.94 ± 0.06	NA	NA	NA	NA	NA	NA
C (mg)	11.56 ± 1.06	61.34 ± 4.96	30	177.51	46	235.90	68	333.33
**Minerals**								
K (mg)	215.82 ± 6.44	1146.16 ± 46.70	214	1266.27	146	748.72	244	1196.08
Na (mg)	30.70 ± 1.87	162.94 ± 8.16	1	5.92	14	71.79	6	29.41
Ca (mg)	18.88 ± 0.23	100.21 ± 0.10	6	35.50	15	76.92	7	34.31
P (mg)	9.81 ± 0.77	52.12 ± 4.66	31	183.43	17	87.17	36	176.47
Mg (mg)	5.25 ± 0.17	27.88 ± 1.21	12	71.01	NA	NA	NA	NA
Fe (mg)	0.24 ± 0.02	1.25 ± 0.09	0.62	3.67	0.5	2.56	0.36	1.76
Zn (mg)	0.20 ± 0.01	1.06 ± 0.06	0.78	4.62	0.13	0.67	0.15	0.74

All data are expressed as mean ± standard deviation (SD) of triplicate experiments (*n* = 3). ^a^ Data per 100 g fresh weight of lychees, rambutans and longans were obtained from the Thai Food Composition Database, food codes: E100, E17 and E98, respectively [27]; ^b^ data per 100 g dry weight of lychees, rambutans and longans were calculated based on the moisture contents of 83.1, 80.5 and 79.6, respectively; ^c^ energy was calculated based on the following equation: energy (kcal) = (protein × 4) + (total carbohydrate × 4) + (fat × 9); ^d^ total carbohydrate was calculated based on the following equation: total carbohydrate (g) = 100 − moisture content (g) − protein (g) − fat (g) − ash (g). DW: dry weight; FW: fresh weight; TDF: total dietary fiber; SDF: soluble dietary fiber; IDF: insoluble dietary fiber; DEF: dietary folate equivalents; NA: data not available.

**Table 3:** Organic acid profile of the aril part of *Nephelium hypoleucum* Kurz.

Organic Acids	Content (g)	
	Per 100 g Fresh Weight	Per 100 g Dry Weight
Oxalic acid	6.64 ± 0.58	1.25 ± 0.11
Formic acid	3.90 ± 0.12	0.73 ± 0.02
Ascorbic acid	1.13 ± 0.06	0.21 ± 0.01
Acetic acid	2.33 ± 0.16	0.44 ± 0.03
Malic acid	0.06 ± 0.00	0.01 ± 0.00
Citric acid	181.16 ± 5.10	34.13 ± 0.96
Succinic acid	ND	ND
Propionic acid	ND	ND

All data are expressed as mean ± standard deviation (SD) of triplicate experiments (*n* = 3). Different superscript letters indicate significantly different contents of different organic acids in the aril of *Nephelium hypoleucum* Kurz (*p* < 0.05) using one-way analysis of variance (ANOVA) and Duncan’s multiple comparison test; ND: not detected.

**Table 4:** Phenolic profiles of different fruit parts of *Nephelium hypoleucum* Kurz.

Bioactive Compounds	Fruit Parts		
	Aril	Pericarp	Seed
**Phenolics (µg/g)**			
Gallic acid	100.19 ± 7.76 ^c^	746.36 ± 52.98 ^b^	2415.32 ± 54.77 ^a^
Rutin	2.78 ± 0.16 ^b^	4.56 ± 0.44 ^a^	2.02 ± 0.13 ^b^
Luteotin	60.42 ± 0.35 ^b^	80.36 ± 0.29 ^a^	ND
Quercetin	366.04 ± 2.90 ^a^	241.79 ± 16.97 ^b^	93.73 ± 4.68 ^c^
Naringenin	0.74 ± 0.01 ^c^	5.52 ± 0.50 ^b^	22.66 ± 0.85 ^a^
Kaempferol	168.19 ± 12.34 ^a^	32.95 ± 0.60 ^b^	ND
Isorhamnetin	4.87 ± 0.31 ^a^	4.59 ± 0.20 ^a^	ND
**TPCs (mg GAE/g DW)**	8.66 ± 0.56 ^c^	18.53 ± 0.81 ^b^	149.45 ± 2.92 ^a^
**TFCs (mg QE/g DW)**	0.56 ± 0.02 ^b^	0.17 ± 0.01 ^c^	0.72 ± 0.07 ^a^
**TACs (mg C3GE/g DW)**	ND	0.21 ± 0.01^a^	ND

All data are expressed as mean ± standard deviation (SD) of triplicate experiments (*n* = 3). Different superscript letters indicate significantly different contents of phenolics in different fruit parts (*p* < 0.05) using one-way analysis of variance (ANOVA) and Duncan’s multiple comparison test; TPCs: total phenolic contents; TFCs: total flavonoid contents; TACs: total anthocyanin contents; GAE: gallic acid equivalent; QE: quercetin equivalent; C3GE: cyanidin-3-O-glucoside equivalent; DW: dry weight; ND: not detected.

**Table 5:** Antioxidant activities of different fruit parts of *Nephelium hypoleucum* Kurz.

Antioxidant Activities	Fruit Parts		
	Aril	Pericarp	Seed
DPPH radical scavenging assay (µmol TE/100 g DW)	1.52 ± 0.04 ^c^	3.35 ± 0.09 ^b^	33.21 ± 0.78 ^a^
FRAP assay (µmol TE/g DW)	75.99 ± 5.89 ^c^	194.01 ± 9.56 ^b^	1307.59 ± 68.51 ^a^
ORAC assay (µmol TE/g DW)	129.37 ± 10.89 ^c^	568.75 ± 35.63 ^b^	741.79 ± 54.30 ^a^

All data are expressed as mean ± standard deviation (SD) of triplicate experiments (*n* = 3). Different superscript letters indicate significantly different contents of antioxidant activities of the same detection method in different fruit parts (*p* < 0.05) using one-way analysis of variance (ANOVA) and Duncan’s multiple comparison test; DPPH: 2,2-diphenyl-1-picrylhydrazyl; FRAP: ferric ion reducing antioxidant power; ORAC: oxygen radical absorbance capacity; TE: Trolox equivalent; DW: dry weight.

**Table 6:** Enzyme inhibitory activities of different fruit parts of *Nephelium hypoleucum* Kurz.

In Vitro Health Properties (% Inhibition)		Fruit Parts		
		Aril	Pericarp	Seed
Obesity	Lipase ^1^	51.59 ± 4.97 ^b^	59.48 ± 3.44 ^a^	ND
Diabetes	α-Amylase ^1^	68.57 ± 6.29 ^a^	35.05 ± 2.78 ^b^	36.11 ± 3.58 ^b^
	α-Glucosidase ^2^	81.19 ± 2.98 ^b^	84.32 ± 5.30 ^b^	93.20 ± 6.53 ^a^
	DPP-IV ^2^	17.14 ± 1.17 ^b^	ND	39.66 ± 2.00 ^a^
Alzheimer’s disease	AChE ^1^	51.33 ± 1.25 ^b^	59.65 ± 1.03 ^b^	84.56 ± 2.87 ^a^
	BChE ^1^	49.66 ± 0.85 ^b^	32.57 ± 2.09 ^c^	90.87 ± 2.63 ^a^
	BACE-1 ^1^	ND	ND	66.33 ± 5.60 ^a^
Hypertension	ACE ^3^	70.35 ± 6.48 ^b^	65.79 ± 4.37 ^b^	85.18 ± 5.33 ^a^

All data are expressed as mean ± standard deviation (SD) of triplicate experiments (*n* = 3). Different superscript letters indicate significantly different inhibitory activities of the same enzyme assay in different fruit parts (*p* < 0.05) using one-way analysis of variance (ANOVA) and Duncan’s multiple comparison test; DPP-IV: dipeptidyl peptidase-IV; AChE: acetylcholinesterase; BChE: butyrylcholinesterase; BACE-1: β-secretase; ACE: angiotensin-converting enzyme; ND: not detected; ^1^ final extract concentration = 8 mg/mL; ^2^ final extract concentration = 1 mg/mL; ^3^ final extract concentration = 0.8 mg/mL.

## Data Availability

Data are contained within this article.

## Supplementary Materials

The following supporting information can be downloaded at: https://www.mdpi.com/article/10.3390/nu15040950/s1, Figure S1: The high-performance liquid chromatograms of (A) organic acid standards and (B) the fruit sample; Figure S2: The liquid chromatography-electrospray ionization tandem mass spectrometry (LC-ESI-MS/MS) chromatograms of (A) phenolic standards and the fruit samples including (B) aril, (C) pericarp and (D) seed; Table S1: Colors of fresh and dry fruits (aril, pericarp and seed) analyzed a ColorFlex EZ spectrophotometer; Table S2: The moisture content of dry fruits (aril, pericarp and seed) analyzed by a Halogen HE53 moisture analyzer.
