# Supplementary material for: Nutrients, Phytochemicals and In Vitro Disease Prevention of Nephelium hypoleucum Kurz Fruit
Source: Nutrients. 2023 Feb 14;15(4):950. doi: 10.3390/nu15040950 (PMC9960393; doi:10.3390/nu15040950)

nutrients

MDPI

Supplementary materials

Nutrients, Phytochemicals and In Vitro Disease Prevention of 4 Nephelium hypoleucum Kurz Fruit

Supplementary Table S1:

Colors of fresh and dry fruits (aril, pericarp and seed) analyzed by a ColorFlex EZ spectrophotometer.

**Fruit parts**	**Color analysis of fresh sample**			**Color analysis of dry sample**		
	**L***	**a***	**b***	**L***	**a***	**b***
Aril	66.45 ± 0.78	0.40 ± 0.12	16.34 ± 0.67	43.38 ± 0.06	4.61 ± 0.04	15.29 ± 0.11
Pericarp	27.19 ± 2.32	26.00 ± 1.19	20.11 ± 1.81	40.35 ± 0.13	5.73 ± 0.02	11.59 ± 0.09
Seed	39.54 ± 1.15	11.47 ± 0.86	24.23 ± 0.86	42.87 ± 0.21	4.63 ± 0.02	18.98 ± 0.08

All data are expressed as mean ± standard deviation (SD) of triplicate experiments (n = 3); L* representing dark (0) to white (100) colors, a* representing green (-) to red (+) colors, and b* representing blue (-) to yellow (+) colors.

Supplementary Table S2:

The moisture content of dry fruits (aril, pericarp and seed) analyzed by a Halogen HE53 moisture analyzer.

**Fruit parts**	**Moisture content of dry sample (%)**
Aril	3.31 ± 0.11
Pericarp	4.81 ± 0.22
Seed	4.61 ± 0.17

All data are expressed as mean ± standard deviation (SD) of triplicate experiments (n = 3).

Supplementary Figure S1:

The high-performance liquid chromatograms of (A) organic acid standards and (B) the fruit sample.

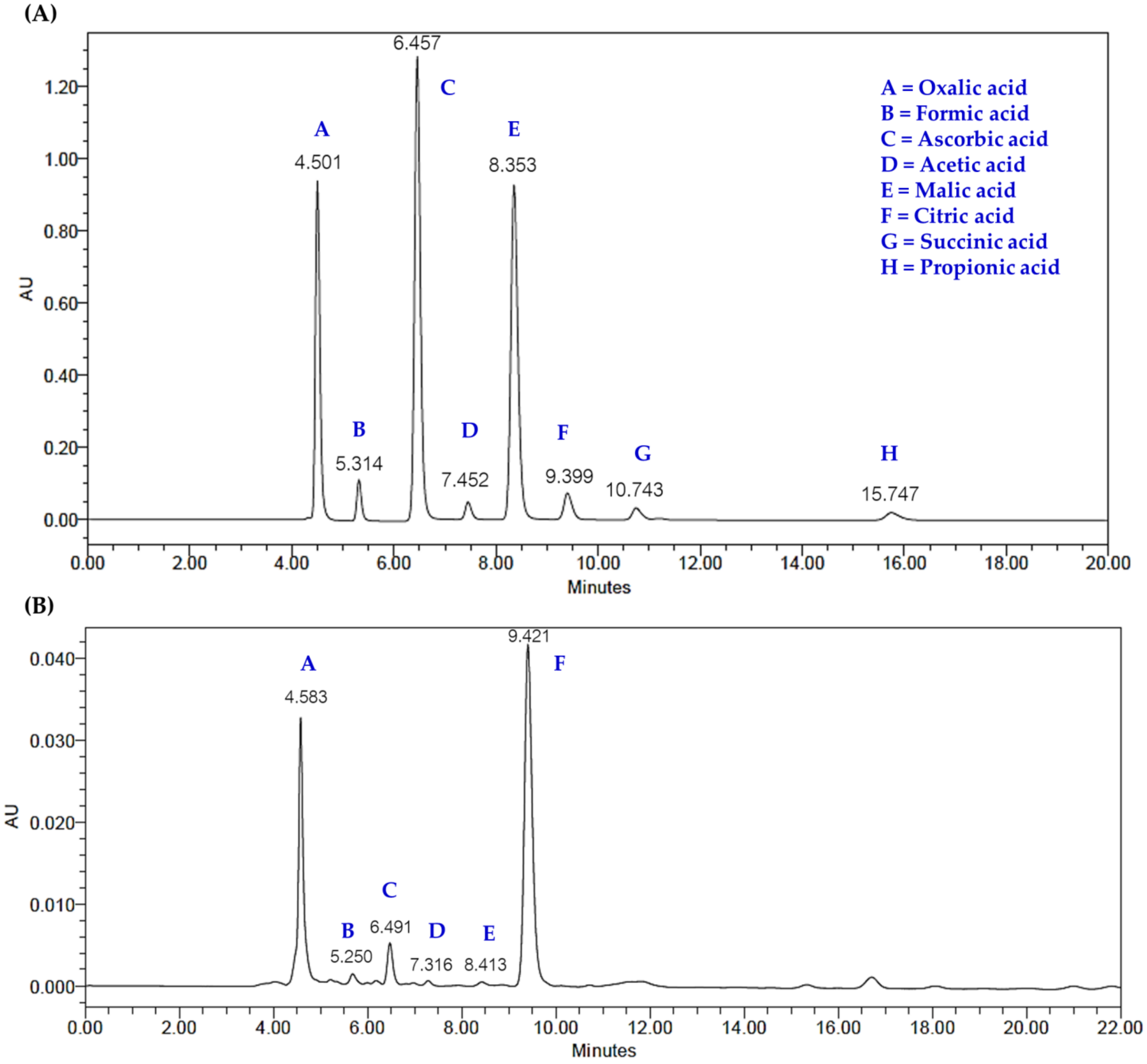

Supplementary Figure S2:

The liquid chromatography-electrospray ionization tandem mass spectrometry (LC-ESI-MS/MS) chromatograms of (A) phenolic standards and the fruit samples including (B) aril, (C) pericarp and (D) seed.

(A)

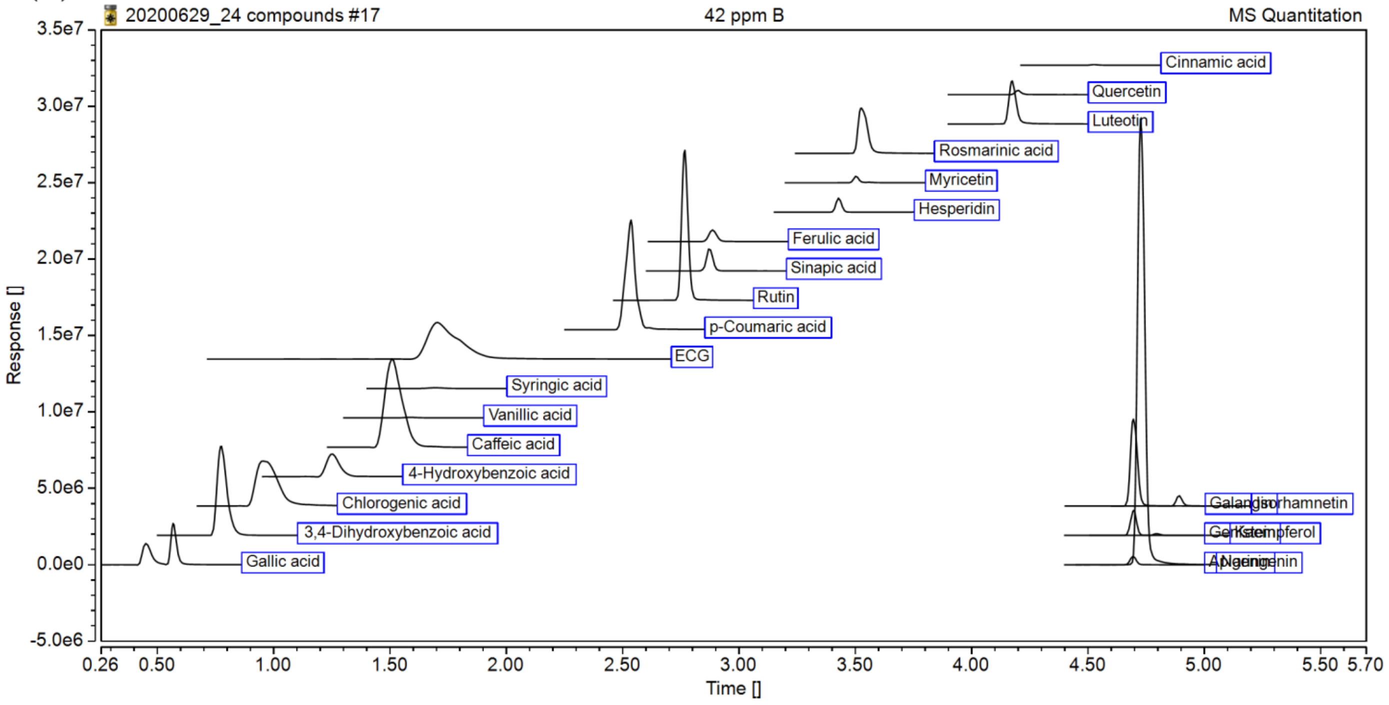

(B)

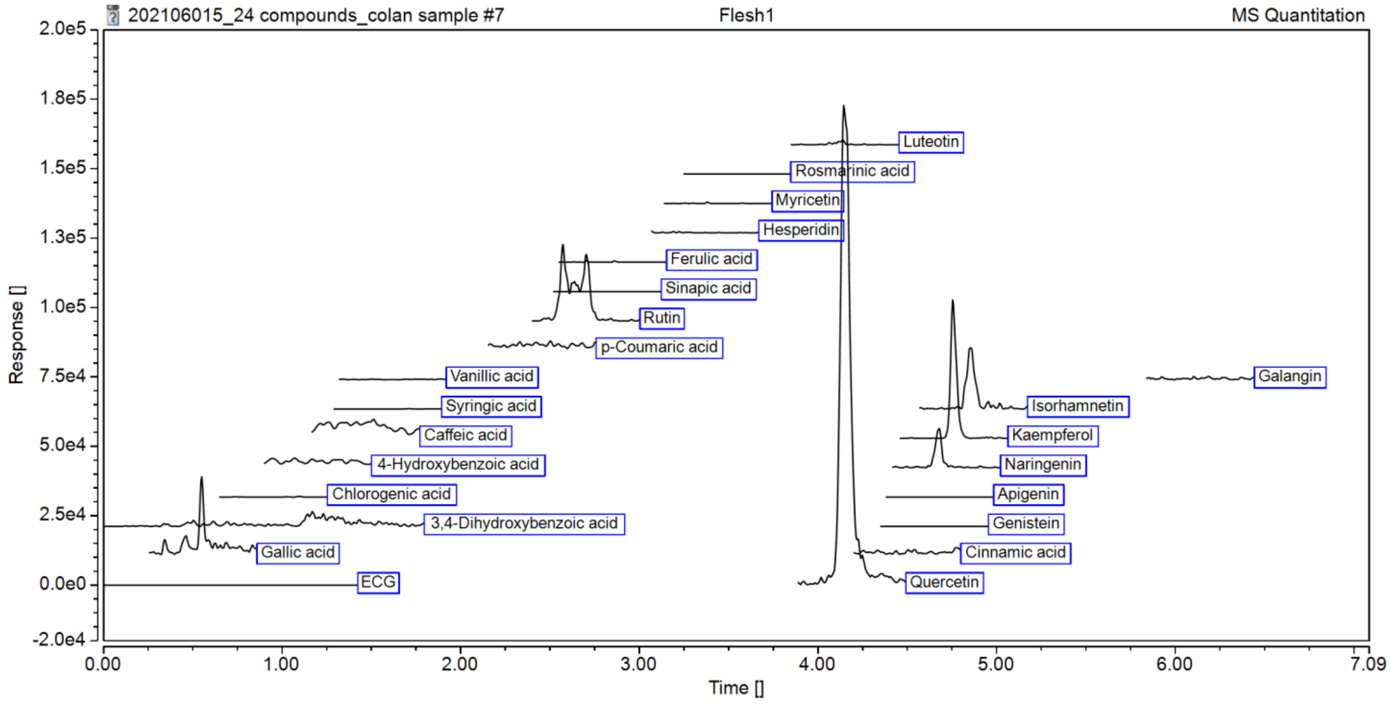

Supplementary Figure S2 (Cont.):

The liquid chromatography-electrospray ionization tandem mass spectrometry (LC-ESI-MS/MS) chromatograms of (A) phenolic standards and the fruit samples including (B) aril, (C) pericarp and (D) seed.

(C)

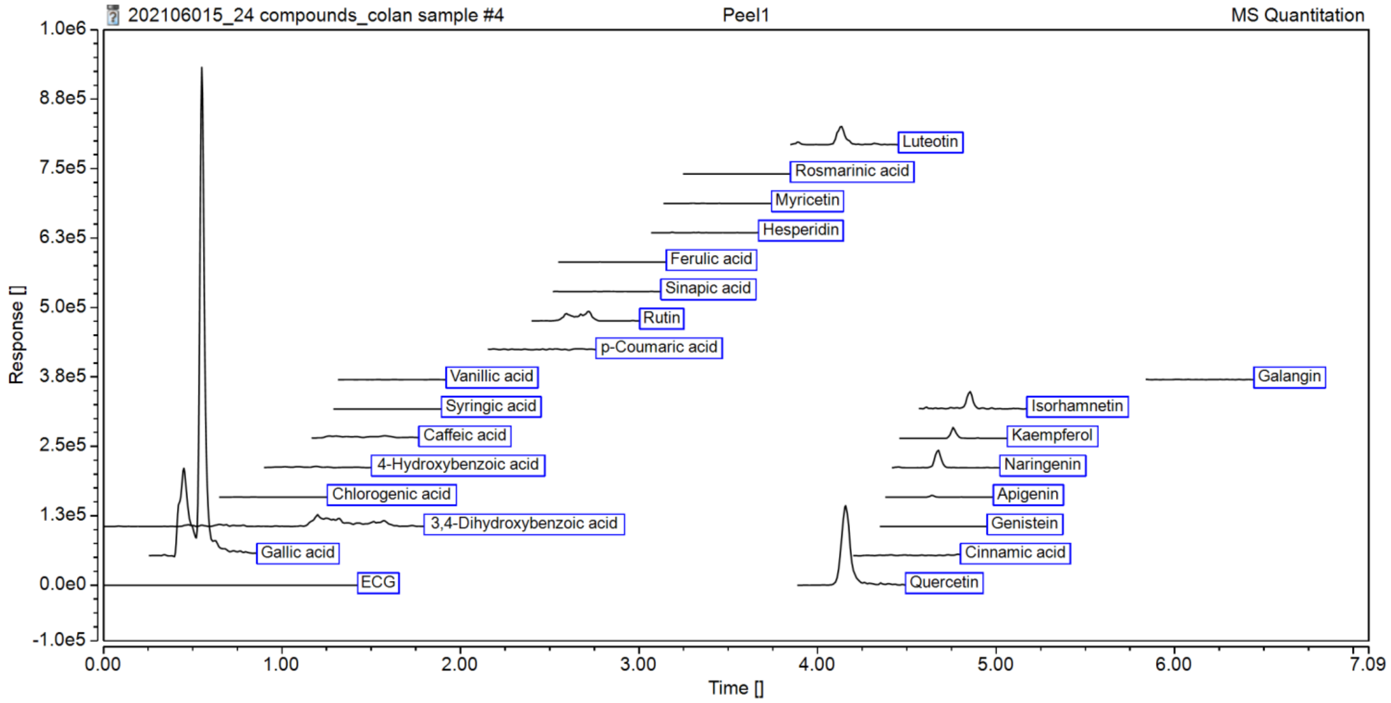

(D)

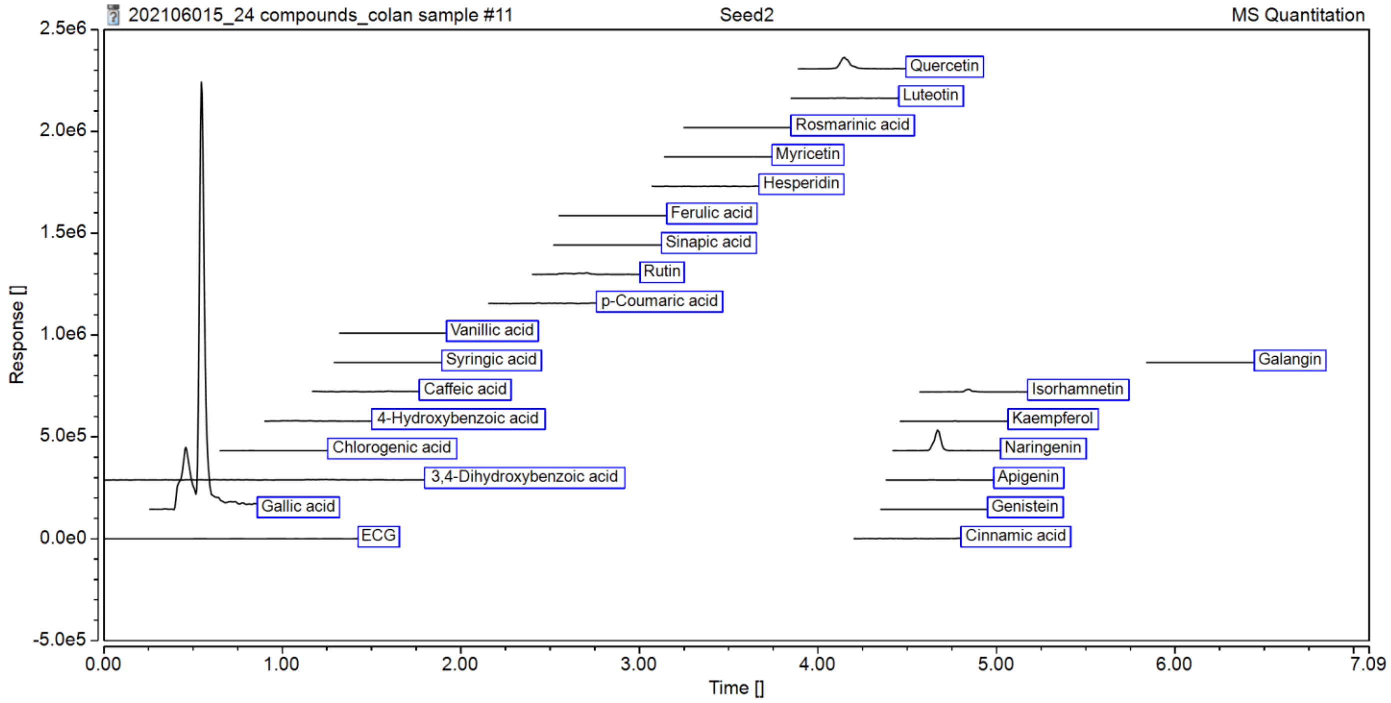

Supplement: Supplementary file 1 [file nutrients-15-00950-s001.zip › nutrients-2198686- supplementary.pdf]
